# Prevalence of chronic hepatitis B and status of HBV care among rural women who planned to conceive in China

**DOI:** 10.1038/s41598-017-12005-2

**Published:** 2017-09-21

**Authors:** Yuanyuan Wang, Hong Zhou, Long Zhang, Qiuyue Zhong, Qiaomei Wang, Haiping Shen, Man Zhang, Yanjie Huang, Anqi Wang, Kenrad Nelson, Yiping Zhang, Donghai Yan, Zuoqi Peng, Ya Zhang, Xiaona Xin, Hongguang Zhang, Jun Zhao, Yan Wang, Ying Yang, Yuan He, Jihong Xu, Xiaoli Liu, Yan Wang, Xu Ma

**Affiliations:** 1 National Research Institute for Family Planning, Beijing, China; 2 Graduate School of Peking Union Medical College, Beijing, China; 3 Environmental and Spatial Epidemiology Research Center, National Human Genetic Resources Center, Beijing, China; 4 Department of Child, Adolescent and Women’s Health, School of Public Health, Peking University, Beijing, China; 5 Department of Epidemiology, Johns Hopkins Bloomberg School of Public Health, Baltimore, Maryland USA; 6 Department of Epidemiology, Harvard T.H. Chan School of Public Health, Boston, Massachusetts, USA; 7 Department of Maternal and Child Health, National Health and Family Planning Commission of the PRC, Beijing, China; 8 Center for Clinical Laboratory, Beijing Shijitan Hospital, Capital Medical University, Beijing, China; 9 Department of Health Policy and Management, Johns Hopkins Bloomberg School of Public Health, Baltimore, Maryland USA

## Abstract

Globally, one third of prevalent chronic hepatitis B (CHB) virus infection (HBV) occurred in China. Assessing the prevalence of CHB infesction and status of HBV-related services among pre-conception women will provide insight into risks of mother to child transmission (MTCT). A cross-sectional analysis of data from the National Free Pre-pregnancy Checkups (NFPC) program in 2010–2014 was conducted. A standardized questionnaire which collected demographic information and enzyme-linked immunosorbent assays (ELISA) which tested serological HBV markers were applied. A total of 16,051,850 rural women aged 15–49 years were included. 5.2% of women were infected with CHB, among whom, 28.6% were also hepatitis B e antigen (HBeAg) positive. The most CHB concentrated places were distributed in southeastern coastal provinces. Women born after 1992 did not experience a higher level of vaccine-induced immunity compared to those born before 1992. Nine in ten rural women with CHB were not aware of their HBV status and a very small proportion of women (0.22%) had received antiviral treatment. Our data demonstrated an overall high-intermediate burden of CHB. Heterogeneity of geographic distribution, high proportion of HBeAg, insufficient awareness of HBV status, and low access to HBV treatment are challenges for preventing the MTCT.

## Introduction

Chronic hepatitis B (CHB) virus (HBV) infection remains a severe problem worldwide especially in China^1^. An estimated 240–400 million persons had chronic HBV infection and the annual mortality from HBV-related complications including cirrhosis and hepatocellular carcinoma was 600,000 persons^2,3^. The most recent national sero-epidemiological survey in 2006 reported 7.2% of CHB in the Chinese population with an estimated 90 million people with CHB^4^, which accounted for 22–38% of the global burden of CHB. Evaluating the prevalence of CHB in key populations in China is critical to inform better prevention policies. The Chinese government recommended routine hepatitis B immunization for newborns in 1992, included it in the National Immunization Program as a free vaccine in 2002 and removed all of the administration fees in 2005^5^. Investigating the prevalence of CHB would also help to evaluate the potential impact of this national HBV vaccination policy.

Rural women in China often had poor education and limited resources^6^ and were likely to resemble populations from other low and middle income countries in terms of education and available resources. Knowing their status of CHB and status of hepatitis B-related services will have important implications onglobal control of CHB. Worldwide, most of CHB has been acquired perinatally or in early childhood^7^, and up to 50% of new HBV infections were obtained through mother-to-child-transmission (MTCT) in China^8^. Identifying women with CHB prior to their pregnancy and gaps of policy implementation are crucial for the prevention of MTCT.

In this study, we reported the prevalence of CHB, geographic distribution and status of hepatitis B-related services among 16 million rural women aged 15–49 years in China, evaluated the effect of the national HBV immunization program on serological outcome in women born before and after 1992 and main focused on challenges on mother to child transmission of HBV.

## Methods

### Study design and study population

A cross-sectional study was conducted, using data from the National Free Pre-pregnancy Checkups (NFPC) program. Women aged 15–49 years who planned to conceive and attended NFPC between January 2010 and December 2014 were included. It is noted that a previous report using 2014 data has demonstrated the burden of HBV in this population. Here we used expanded samples from 2010–2014 to see if the conclusions remain and to explore other prioritized questions^9^. The NFPC, supported by the Chinese National Health and Family Planning Commission and Ministry of Finance, is a nationwide free preconception medical examination service for rural couples in China. It was first carried out in 100 counties of 18 provinces in 2010 in China, and was gradually extended to 2790 counties in 2013 with high coverage across the country. Since the NFPC is a free program aiming to improve the health of pregnancy and reduce birth defects, and women voluntarily came to obtain the service, the selection of study population would belong to the convenience sampling.

All couples who planned to conceive were enrolled by trained staff, and informed consent was obtained before the checkups. A face-to-face interview was then carried out by qualified nurses using a standardized procedure. Socioeconomic information including age, occupation, education, ethnicity, and residence was collected by a structured questionnaire. A 5 mL-blood sample was obtained and tested for HBV serologic markers at local laboratories. Tests included hepatitis B surface antigen (HBsAg), hepatitis B surface antibody (anti-HBs), hepatitis B e antigen (HBeAg), hepatitis B e antibody (anti-HBe) and hepatitis B core antibody (anti-HBc) using ELISA kits approved by the China Food and Drug Administration. Quality control was completed by the National Center of Clinical Laboratories with reagents produced by Abbott (Abbott Park, IL, USA), as the reference standard. Study protocols and forms were approved by the Institutional Research Review Board at the National Research Institute for Family Planning. The study was carried out under the guidance of the protocols strictly.

### Definition of outcomes

The primary outcomes included the prevalence of chronic HBV infection, defined as positivity of HBsAg (less commonly indicative of acute infection)^2^, self-reported awareness of HBV status, and initiation of antiviral therapy against HBV. Secondary outcomes comprised past/present HBV infection (positivity of anti-HBc), high infectivity (positivity of HBeAg) and vaccine-induced immunity (isolated positivity of anti-HBs).

### Definition of covariates

Participants’ characteristics included: age (15–19, 20–24, 25–29, 30–34, 35–39, 40–44 and 45–49 years) education level (illiterate, primary school, junior school, senior school and college or higher), ethnic group (Han and others), migrant population (yes, no), region of residence (eastern, central and western)^10^, a self-reported history of HBV immunization (yes, no) and parity (0, ≥ 1). To explore the impact of the national HBV immunization program beginning in 1992, study population were divided into a cohort born before 1992 and a cohort born after 1992.

### Statistical analysis

We present the distribution of participants’ socio-demographic characteristics using proportions. The prevalence of each outcome and 95% confidence interval (CI) were calculated in the entire population, as well as in subgroups. We employed Poisson regression models to assess the crude and adjusted prevalence ratio (PR) of outcomes. Adjusted variables for awareness of HBV status were age, education, ethnic group, occupation, migrant population, region and parity. The effect of the national HBV immunization program was analyzed by comparing the difference of each serological outcome in women born before and after 1992. In addition, to describe the geographical distribution, we estimated the prevalence of CHB in each of 2,421 counties after exclusion of counties with no data or sample size less than 100 (assume unstable), and then county-specific prevalence of HBsAg was mapped in four tiers (low: <2%, low intermediate: 2–4%, high intermediate: 5–8% and high: >8%) using ArcGIS 10.3 (ESRI, Redlands, California, USA).A two-tailed p value < 0.05 was considered to reach statistical significance. SPSS version 20.0 (IBM Inc., Armonk, NY) and Stata 13.1 (College Station, Texas, USA) were used for all analyses.

## Results

**Figure 1:**
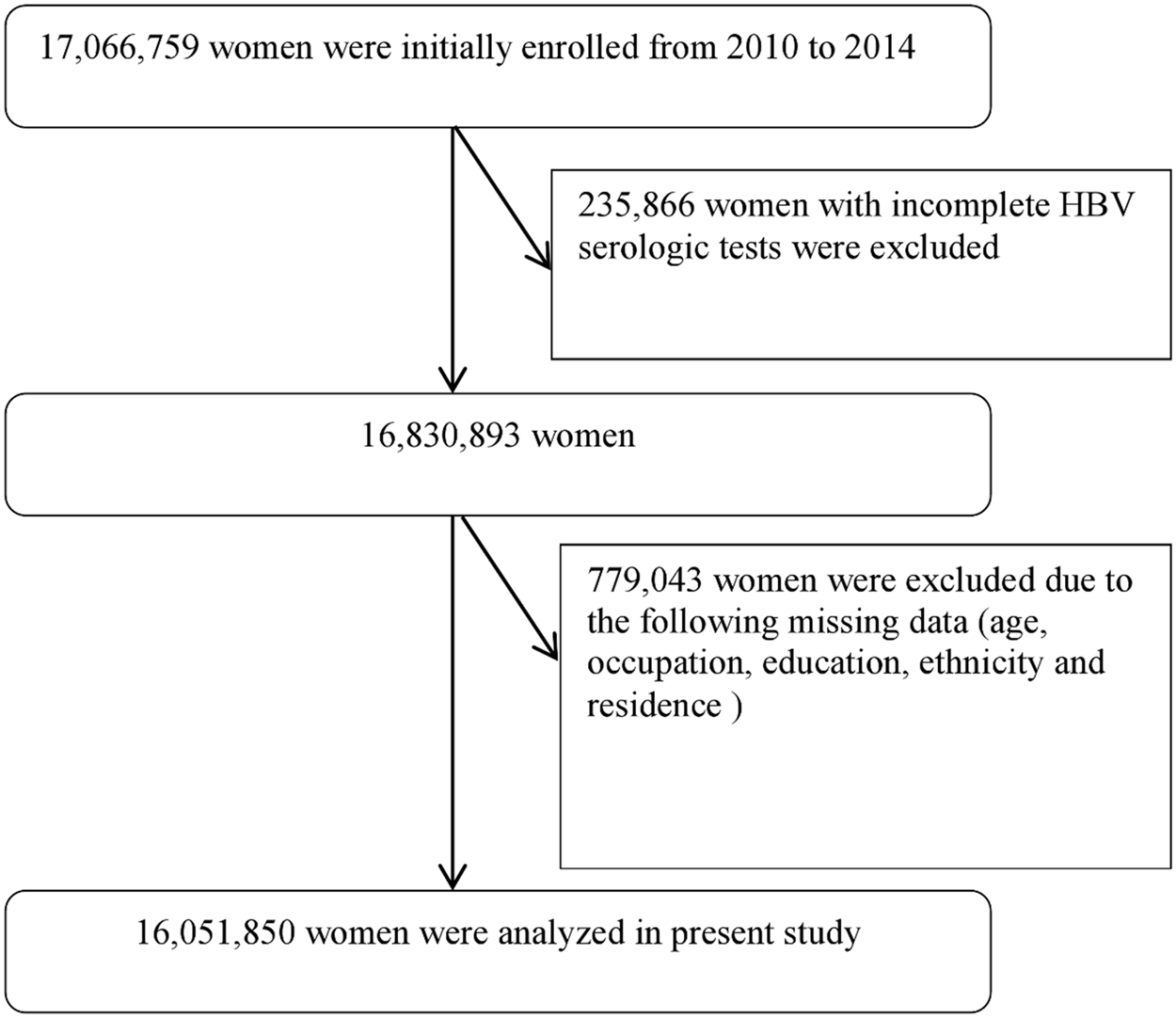
Flowchart of the study.

Figure 1 presents a flowchart of the study population and analytical scheme. Between 2010 and 2014, a total of 17,066,759 women were recorded in the NFPC program. 16,830,893 (98.6%) of them completed HBV serological testing, and 16,051,850 (94.1%) women with complete demographic information were included in the analysis. The majority of 16,051,850 women were aged 20–29 years (77.3%), Han ethnicity (88.1%), farmers (77.5%), and non-migrant population (96.6%), and had education of junior school (65.0%). Locations where participants currently live were approximately evenly distributed across the eastern (33.9%), central (37.6%) and western (28.5%) regions. 26.8% of women reported a history of HBV immunization (Table 1).

**Table 1:** Characteristics of Study Population.

	**Total**
Overall	16,051,850
Age	
15–19	260,705 (1.6%)
20–24	6,467,204 (40.3%)
25–29	5,942,888 (37.0%)
30–34	2,213,776 (13.8%)
35–39	820,370 (5.1%)
40–44	300,939 (1.8%)
45–49	45,968 (0.3%)
Education	
Illiterate	68,080 (0.4%)
Primary school	873,307 (5.4%)
Junior school	10,428,434 (65.0%)
Senior school	2,675,795 (16.7%)
College or higher	2,006,234 (12.5%)
Ethnic group	
Han	14,140,650 (88.1%)
Others	1,911,200 (11.9%)
Occupation	
Farmers	12,438,133 (77.5%)
Others	3,613,717 (22.5%)
Migrant Population	
Yes	541,071 (3.4%)
No	15,510,779 (96.6%)
Region	
Eastern	5,439,043 (33.9%)
Central	6,030,488 (37.6%)
Western	4,582,319 (28.5%)
A Self-reported History of HBV Immunisation	
Yes	4,295,344 (26.8%)
No	11,756,506 (73.2%)

**Figure 2:**
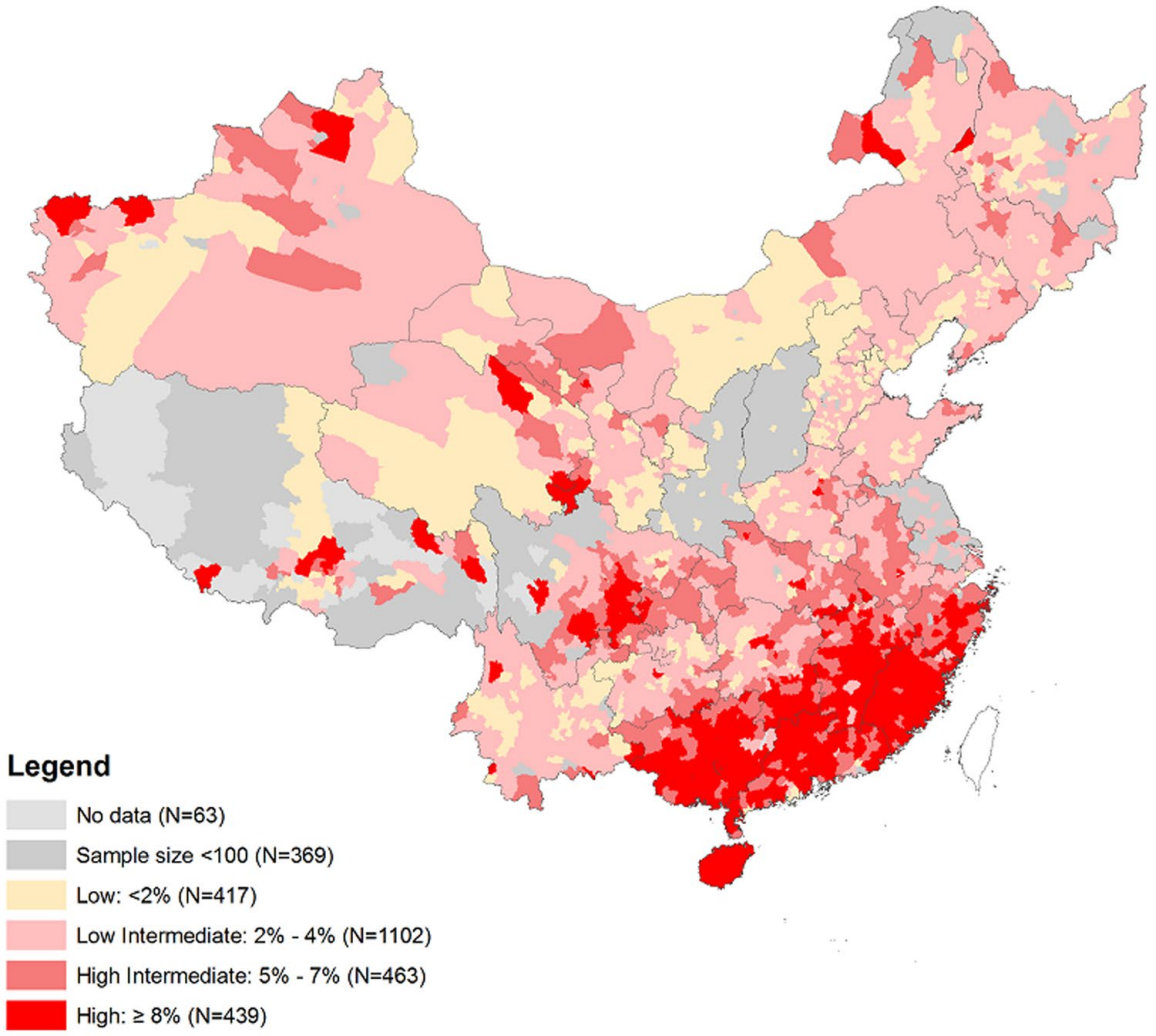
The prevalence of chronic hepatitis B in different geographic regions. The Fig. 2 was created using ArcGIS 10.3 (ESRI, Redlands, California, USA). We used the China 2010 Census GIS shapefile as the basic layer, and linked it with the count-level prevalence of HBsAg, collected by National Free Pre-conception Check-up (NFPC) program, by county name. Then the burden of HBsAg was displayed into predefined four tiers with four different colors. The China 2010 Census GIS shapefile is derived from the China Data Center production: 2010 China County Population Census Data with GIS Maps (Version I). This dataset is in GBK encoding. Publication date: May 2013. Harvard Acquisition date: Jan 2014. http://hollis.harvard.edu/primo_library/libweb/action/dlDisplay.do?vid=HVD&search_scope=default_scope&docId=HVD_ALEPH013684533&fn=permalink.

The crude prevalence of CHB among all women was 5.2% and age-adjusted prevalence based on population weight from China 2010 Census Data is 5.0% (Table 2). The prevalence of HBeAg among women with CHB was 28.6%. Of the total 2,421 counties across China, there are 439 (18.1%) high, 463 (19.1%) high-intermediate, 1102 (45.5%) low-intermediate, and 417 (17.2%) low CHB endemic counties, and the top 10 county-specific prevalence of HBsAg ranged from 17.6% to 22.4%. The majority of high endemic counties were concentrated in five provinces including Guangxi, Guangdong, Fujian, Hainan and Jiangxi (Fig. 2). Women who were born after 1992 had a lower prevalence of CHB (4.7% vs 5.3%), past/present HBV infection (8.3% vs 10.5%), and vaccine-induced immunity (26.2% vs 29.3%) compared to those born before1992 Table 3).

**Table 3:** Prevalence of Each HBV Serological Outcome by Selected Characteristics in China

	**Born before 1992 (N = 14,605,760)**			**Born after 1992 (N = 1,446,090)**			Crude PR* (95% CI)		Adjusted PR* (95% CI)	
	**n**	**%(95% CI)**		**n**	**%(95% CI)**					
**HBsAg**	768864	5.3	(5.3–5.3)	68508	4.7	(4.7–4.8)	0.89	(0.89–0.90)	0.91	(0.91–0.92)
**Anti-HBc**	1528618	10.5	(10.5–10.5)	120002	8.3	(8.3–8.3)	0.79	(0.78–0.79)	0.82	(0.81–0.83)
**Anti-HBs**	5022547	34.4	(34.4–34.4)	432292	29.9	(29.8–30.0)	0.87	(0.86–0.87)	0.94	(0.94–0.95)
**Isolated Anti-HBs**	4272538	29.3	(29.2–29.3)	378696	26.2	(26.1–26.3)	0.89	(0.89–0.89)	0.98	(0.97–0.98)

Abbreviations: HBsAg, hepatitis B surface antigen; anti-HBs, hepatitis B surface antibody; isolated anti-HBs refers to only anti-HBs positive; anti-HBc, hepatitis B core antibody.

*Women born before 1992 are the reference group.

**Table 4:** Prevalence of Awareness of HBV Status among Women with CHB in China

	**CHB, N**	**Awareness, n**	**% (95% CI)**	**Crude PR**	**Adjusted PR**
Overall	837372	85725	10.2(10.2–10.3)		
Age					
15–19	9365	480	5.1(4.7–5.6)	0.43(0.39–0.47)	0.59(0.54–0.65)
20–24	332220	30196	9.1(9.0–9.2)	0.76(0.75–0.77)	0.89(0.87–0.90)
25–29	313734	37184	11.9(11.7–12.0)	REF	REF
30–34	118139	13118	11.1(10.9–11.3)	0.93(0.91–0.95)	0.92(0.90–0.93)
35–39	45757	3792	8.3(8.0–8.5)	0.69(0.67–0.72)	0.71(0.68–0.73)
40–44	15906	865	5.4(5.1–5.8)	0.45(0.42–0.48)	0.49(0.46–0.53)
45–49	2251	90	4.0(3.2–4.8)	0.33(0.27–0.41)	0.38(0.31–0.46)
Education					
Illiterate	4531	313	6.9(6.2–7.6)	0.40(0.36–0.45)	0.46(0.41–0.51)
Primary school	47751	3507	7.3(7.1–7.6)	0.43(0.41–0.44)	0.47(0.45–0.48)
Junior school	534603	45321	8.5(8.4–8.6)	0.49(0.48–0.50)	0.56(0.55–0.57)
Senior school	144020	18411	12.8(12.6–13.0)	0.74(0.73–0.76)	0.81(0.79–0.83)
College or higher	106467	18173	17.1(16.8–17.3)	REF	REF
Ethnic group					
Han	743676	76260	10.3(10.2–10.3)	REF	REF
Others	93696	9465	10.1(9.9–10.3)	0.98(0.96–1.00)	1.00(0.98–1.02)
Occupation					
Farmers	616064	52628	8.5(8.5–8.6)	REF	REF
Others	221308	33097	15.0(14.8–15.1)	1.75(1.72–1.77)	1.51(1.49–1.53)
Migrant Population					
Yes	32846	4445	13.5(13.2–13.9)	1.33(1.30–1.37)	1.23(1.19–1.26)
No	804526	81280	10.1(10.0–10.2)	REF	REF
Region					
Eastern	306711	33918	11.1(10.9–11.2)	0.97(0.95–0.98)	0.77(0.75–0.78)
Central	301156	25687	8.5(8.4–8.6)	0.74(0.73–0.76)	0.74(0.73–0.76)
Western	229505	26120	11.4(11.3–11.5)	REF	REF
Parity					
0	435867	42317	9.7(9.6–9.8)	0.90(0.89–0.91)	0.70(0.69–0.71)
≥1	401505	43408	10.8(10.7–10.9)	REF	REF

Abbreviations: crude prevalence ratio (crude PR), adjusted prevalence ratio (adjusted PR), reference (REF).

Of 837,372 women with CHB, only 85,725 (10.2%) were aware of their HBV status. Age 15–24 years and 30–49 years (vs 25–29 years), education of senior school or less (vs college or higher), living in eastern or central region (vs western region) and zero parity (vs ≥ 1) were significantly associated with a lower prevalence of awareness of HBV status (Table 4). 1874 (0.22%) women had received some therapies related to hepatitis B. Using alanine transaminase (ALT) > 2 upper limits of normal (2 ULN = 76 U/L) and HBeAg positivity as criteria for the initiation of treatment for CHB^10,11^, 7.4% (62,343/837,372) women with CHB actually need antiviral therapy for their active HBV infection. The most common antiviral drug used was Telbivudine (16.1%, 301/1,874), followed by Lamivudine (11.8%, 222/1,874), Adefovir (10.7%, 201/1,874), Entecavir (5.3%, 100/1,874), Peg-IFN-2a (2.0%, 37/1,874) and Tenofovir (1.0%, 18/1,874).

**Table 2:** Crude and Age-adjusted Prevalence of CHB infection

Age groups	Number of women in present study	Number of HBsAg positive women	Prevalence of HBsAg	Number of women in China 2015 Census Data	Population weight based on China 2015 Census Data
15–19	260,705	9,365	3.6%	21,998,069	0.13
20–29	12,410,092	645,954	5.2%	49,857,585	0.29
30–39	3,034,146	163,896	5.4%	45,996,267	0.26
40–49	346,907	18,157	5.2%	56,116,285	0.32

Note: Crude prevalence of CHB infection is 5.2%, and age-adjusted prevalence based on population weight from China 2015 Census Data is 5.0%.

## Discussion

This study reports a high-intermediate burden of chronic HBV infection in a large sample of women aged 15–49 years from rural China who planned to conceive. Superimposed on this, the heterogeneity of geographic distribution and low vaccine induced immunity even among women born after 1992 when China began to recommend HBV vaccination, are barriers to prevention of MTCT of HBV infection. Most women (90%) with CHB were not aware of their status, and only a very small proportion of them had a history of antiviral therapy despite a substantial proportion (7.4%) who may qualify, which acts as additional barriers to prevent MTCT. Although CHB can be controlled with timely and careful monitoring and appropriate treatment, these data show that preventive education and access to the clinical care among rural women with CHB are not far from optimum.

The overall prevalence of CHB (5.2%) was lower than 8.7% in 2006^4^ and 9.7% in 1992^12^ in the same age groups, reported in two latest national HBV seros urveys. Also, the prevalence of CHB among rural men from the same data source was higher than rural women (7.0% vs 5.2%)^13^.

Geographically, 16% of provinces and 18% of counties in China still had high endemicity of HBV infection ( >8%) among rural women aged 15–49 years. The most HBV concentrated places are southeastern coastal provinces, including Guangxi, Guangdong, Fujian, Hainan, and Jiangxi. It is hard to distinguish whether individual factors, like low socio-economic status and inter-province migration^14^, or factors related to geographical locations, like historical HBV endemicity and higher risk of sexual transmission in these places, contribute to this aggregation. Besides figuring out contributing factors, preventive efforts, like screening and education, targeting on these clustered locations are recommended.

The Chinese government recommended HBV immunization for newborns in 1992. Because of high prices and unequal access to vaccines in early years^15^, people born before or close to 1992, would not benefit from this policy. Our results showed that even for women born after 1992, either overall immunity or vaccine-induced immunity against HBV did not improve compared to that of women born before 1992. Of note, women who reported a history of HBV immunization had a lower prevalence of CHB (4.4% vs 5.5%) and higher proportion of overall immunity (41.4% vs 31.3%) versus those without a self-reported history of HBV immunization. This reminds that although women missed their immunization at birth or early childhood, being vaccinated later in adulthood may still be protective.

The high proportion of positivity of HBeAg, an indication of high infectivity^16^, among women with chronic HBV infection could facilitate the risk of MTCT. Although administration of hepatitis B vaccination in conjunction with HBIG among newborns is highly effective to prevent transmission^7^,about 7% of infants born to mothers with high viral load, indicated by positivity of HBeAg, could still be infected with HBV^17^. Our data showed that 29% of rural women with CHB were also HBeAg positive. It means not only is the burden of CHB high, but women infected with CHB are at higher risk of transmitting the disease to others, like their future babies.

As outlined by WHO in 2015, in the management of chronic HBV infection^7^, being tested and diagnosed is the first step to link people to care. Those subjects with active hepatic inflammation should be linked to care and evaluated for antiviral therapy. Our results showed that nine in ten rural women with chronic HBV infection were not aware of their status and a very small proportion of women had received treatment (0.22%) despite more women in need of antiviral therapy (7.4%). A recent study showed that Tenofovir regimen in pregnant women during their pregnancy in conjunction with a standard newborn immunoprophylaxis could significantly decrease the rate of MTCT compared to group who only had a newborn immunoprophylaxis^17^. This suggests that decreasing viral load to a suppressed level among pregnant or prenatal women through antiviral treatment is key to add further protection from MTCT. However, rural women with CHB in this study had a very low rate of receiving antiviral treatment, which makes it impossible to suppress their viral load to a safe level and prevent MTCT effectively.

The data includes a very large sample of rural women who planned to conceive, who represent a critical reservoir for the continued MTCT of HBV in China. Also, this is the first study to evaluate the status of HBV care among rural women in China, which can inform better prevention and treatment service delivery. Limitations include inability of generalizing results to the entire population since the study population comprised only rural women from a convenience sample; a potential selection bias as women who participated in the study may differ from those who had not participated; lack of quantification of viral load and anti-HBs titer; lack of continuous monitoring to confirm eligibility of antiviral treatment; a recall bias of self-reported awareness of HBV status and antiviral treatment experience.

In conclusion, our data demonstrate a high-intermediate burden of chronic HBV infection in China. The heterogeneity of geographic distribution, high proportion of HBeAg, insufficient awareness of HBV status, and low rate of antiviral treatment are deteriorating health among rural women with CHB and they will also be reservoirs for the continued MTCT of HBV in China. Public health efforts to improve awareness of HBV, to provide a catch-up vaccination and to increase uptake of appropriate antiviral treatment should be scaled up immediately.
